# 

**Published:** 1881-08

**Authors:** 


					﻿Whole Number,
CCCCXXXIX.
August, 1881.
Number 2,
Vol. XLIII.
THE
CHICAGO
MEDICAL
T ournal and Examiner
EDITORS:
N. S. DAVIS, M.D., LL.D. JAS. NEVINS HYDE, A.M., M.D.
DANIEL R. BROWER, M.D.
CHICAGO:
PUBLISHED BY THE CHICAGO MEDICAL PRESS ASSOCIATION,
188'Clark Street.
^Read the Notice of this Journal among Advertisements.
				

